# Drug Persistence of Patients with Inflammatory Bowel Disease Under Biological Treatment in the Pre IL-23 Era in a Tertiary Referral Center in Germany

**DOI:** 10.3390/jcm14248918

**Published:** 2025-12-17

**Authors:** Karima Farrag, Lars Grimm, Iulia Dahmer, Katharina Stratmann, Antje Dienethal, Kathrin Sprinzl, Raul Lande, Stefan Zeuzem, Irina Blumenstein, Alica Kubesch

**Affiliations:** 1 Medical Clinic 1, University Hospital, Goethe University Frankfurt, 60590 Frankfurt am Main, Germany; 2 Institute of Biostatistics and Mathematical Modelling, Goethe University Frankfurt, 60590 Frankfurt am Main, Germany

**Keywords:** IBD, Crohn’s disease, ulcerative colitis, biologics, real-world data, drug persistence

## Abstract

**Background/Objectives:** Predicting treatment persistence in inflammatory bowel disease (IBD) remains challenging despite a broadened therapeutic arsenal. This study used longitudinal data to assess drug persistence across four biologics for IBD prior to Interleukin (IL)-23 blocker approval. **Methods:** We retrospectively analyzed IBD outpatients at Goethe University Hospital. Laboratory data and treatment adherence were collected to determine how many patients continued each biological therapy after induction and at 1 year. **Results:** Of 587 patients, those on azathioprine or mercaptopurine were excluded (focusing on biologics). Four biologicals were analyzed: 280 patients received one of them; 312 (53.2%) had Crohn’s disease and 275 (46.8%) had Ulcerative Colitis. Infliximab (IFX) was given to 93 patients (median 912 days; range 20–5273); at endpoint, 39 (42.4%) remained on IFX. Adalimumab (ADA) was used by 165 patients (median 1051 days; range 48–4458); 87 (52.4%) remained at endpoint. Vedolizumab (VDZ) included 116 patients (median 717 days; range 0–2204); 75 (64.4%) remained at endpoint. Ustekinumab (UST) was given to 62 patients (median 660 days; range 50–1399); 51 (83.6%) remained at endpoint. Some patients were exposed to multiple biologicals, contributing to smaller numbers for newer drugs. When evaluated as a first-line biological, UST (median 787 days) and VDZ (median 756.5 days) had the shortest durations, while TNF-α blockers (ADA, IFX) showed the greatest resistance as first-line therapy. **Conclusions:** Most patients stayed on their initial therapy after induction and at 1 year. These findings support the sustained use of established biological therapies as cost-effective, steroid-sparing options even after IL-23 approval and may inform patient counseling and long-term pharmacoeconomic considerations.

## 1. Introduction

Inflammatory bowel disease (IBD), comprising ulcerative colitis (UC) and Crohn’s disease (CD), is characterized by recurrent inflammation and a chronic, often progressive, course. Achieving and maintaining remission are central goals in IBD management. As both diseases tend to progress, treatment focus has shifted from sole symptom control to preventing disease progression and long-term complications. Consequently, modern therapies aim to control disease activity, induce and sustain remission, and be suitable for long-term administration with favorable safety profiles.

The first substance group to be approved for the treatment of moderate to severe CD and UC were anti-tumor necrosis factor alpha (TNF alpha) agents [1,2,3,4,5,6,7,8]. Since their introduction, they have proven to be potent in inducing and maintaining remission and to be safe for long-term application [1,2,3,5,6,7,8,9].

The Integrin-antagonist Vedolizumab (VDZ) has joined the treatment armory in 2014 in Germany. Through its localized mode of action—selectively inhibiting gastrointestinal inflammation—this substance class is well tolerated and only few unwanted side effects are reported [10,11,12].

Lastly, the interleukin (IL)-12/23 antagonists were approved for the treatment of first CD in 2016 and later UC in 2019 [13,14].

More recently, IL-23 antagonists have become available for UC and CD. As this study was conducted retrospectively, data analysis ceased before these newer therapies were accessible. Drug safety and efficacy has been proven in several randomized controlled clinical trials and observational studies for all three substance groups [15].

Nonetheless, a relevant percentage of patients fail to respond to first-line therapy.

For anti-TNF, the numbers for first-line treatment failure are as high as 30% and between 25–40% of patients experience a loss of response over time. Comparable treatment failure rates are available for Integrin-antagonists and IL-12/23 inhibitors [16,17,18].

With a broader treatment armamentarium, clinicians and patients now have multiple options and the possibility to switch therapies if needed. In this context, drug persistence becomes increasingly informative, reflecting downstream effects on efficacy and safety and guiding individualized treatment decisions and algorithms. However, data on persistence—especially when distinguishing treatment-naïve from experienced patients—remain incomplete.

Therefore, the present study aimed to determine drug-persistence rates in both biologic-naïve and biologic-experienced patients with CD and UC, to inform personalized therapy decisions and care pathways.

## 2. Patients and Methods

### 2.1. Study Design and Population

In this retrospective study, all patients with CD and UC receiving treatment for their IBD at the outpatient clinic of the University Hospital, Medical Clinic 1, Frankfurt, Germany from 1 January 2017 until 31 March 2020 were included. All patients had to be diagnosed with CD or UC according to standard criteria and had to be ≥18 years. There were no other exclusion criteria. Patients were followed up longitudinally over time and medication switches were recorded.

### 2.2. Study Variables

Patient characteristics included age, gender, disease duration, behavior, and location [19,20] (Vienna Classification and Montreal Classification) [21], previous biological treatment, and biomarkers for disease activity [19,20].

### 2.3. Outcome Measures

The date of treatment initiation was defined as baseline. The primary study objective was to evaluate drug persistence at 12 months on the newly initiated biological treatment. Drug persistence served as a representative for clinical efficacy. Drug persistence was documented for the entire cohort. For each medication the following time-points were evaluated: induction achieved, 3 months on medication, 6 months on medication as well as 1 year on medication. Induction achieved means that the required intravenous administrations have been performed according to the manufacturer’s recommendations, for example, three doses of IFX intravenously before switching to subcutaneous (s.c.) administration. The end of treatment was documented if documentation existed on a stop or switch in medication (i.e., documented clinic visits with respective information or new prescription). Furthermore, we assessed whether patients were previously exposed to a biological agent, and we also assessed disease activity with the help of fecal Calprotectin (fCal) if available. Concomitant steroid use at baseline was also documented. In this cohort no other concomitant therapy was used.

### 2.4. Ethics Statement

Approval for this retrospective study was obtained from the local Ethics Committee of the University Hospital Frankfurt (file number 2022-708).

### 2.5. Statistical Analysis

The data were collected using the electronic medical record in the Patient Management System ORBIS^®^ (Version: 08044303.00040.DACHL). From the collected data, a Microsoft^®^ Excel^®^ table was created that contains the corresponding parameters to be examined.

Data were first analyzed descriptively and, consequently, comparisons between independent groups were performed. Nominal variables were compared using the Chi-squared test, continuous data using the two-sample *t*-test when data were normally distributed and Wilcoxon–Mann–Whitney–U-Tests otherwise. The binomial test was employed for testing for equal proportions in two groups.

We employed linear mixed-effects models (LMMs) to analyze log-transformed longitudinal (repeated-measures) data of the continuous biomarker, calprotectin. The model included time, disease type, and treatment as fixed effects and a random intercept to account for within-subject correlation across repeated measurements. Goodness-to-fit measures were reported.

For comparing the times until a change in medication for the different drugs, a Cox regression with the drug as independent variable was performed. Since the patients underwent several different medications and thus more entries per patient were present in the data, the patient was considered as a cluster in the model. The assumption of proportional hazards was verified, and a robust sandwich-type variance estimator was used. Age, gender, body mass index (BMI), biologic-naïve status, disease duration, the number of previous treatment failures, and administration of steroids at treatment start were included in the regression as confounders. Kaplan–Meier curves were used for graphical representation.

Complete case analyses were performed for the Cox regression and the mixed-effects regression. Missingness in the data was accounted for by performing sensitivity analyses using imputation.

Normality of the data was tested using the Shapiro–Wilk test.

All tests were performed two-sided and *p*-values smaller than 5% were considered as significant.

The analysis was conducted using IBM SPSS Statistics (version 31.0.0.0, https://www.ibm.com/de-de/products/spss-statistics, accessed on 10 June 2025), RStudio (version 2025.09.0+418, https://posit.co/download/rstudio-desktop/, accessed on 11 September 2025), and R (version 4.5.1, https://www.r-project.org/, accessed on 13 June 2025), with the packages “dplyr”, “tidyr”, “survival”, “survminer”, “nlme”, “performance”, “mice”, and “PMCMRplus”.

## 3. Results

### 3.1. Patient Characteristics

In our study we retrospectively investigated 587 patients with previously confirmed IBD. There was no significant difference in number of patients between patients with UC and patients with CD. The percentage of female patients was higher in the CD group (Chi Quadrat Test, *p* = 0.001).

A total of 311 patients were female (53%), and 276 patients were male (47%). The median age was 44 years (range 20–87). Patients with UC were significantly older (Wilcoxon–Mann–Whitney Test, *p* = 0.02).

The median disease duration (n = 576) was 14 years (range 1–52). Patients with CD had a longer disease duration (Wilcoxon–Mann–Whitney Test, *p* < 0.001). Data for eleven patients concerning disease duration were missing. Thus, only 576 patients were included in this analysis. A total of 312 patients (53.2%) suffered from CD and 275 (46.8%) patients suffered from UC (Table 1). Data on disease behavior are provided in Table 1a,b.

Most patients in both anti-TNF groups (i.e., IFX and ADA) were biological-treatment-naïve (IFX 74% vs. ADA 81%) at the start of the investigated treatment. The number of treatment-naïve patients decreased for VDZ (43%) and UST (16%).

### 3.2. Drug Persistence

Drug persistence was assessed for the subcohort of patients who received at least one of the four drugs (n = 280). Patients treated with azathioprine or mercaptopurine were excluded from the analysis, as the study specifically aimed to investigate biologic therapies.

A total of 93 patients were treated with IFX with a median duration of 912 days (20–5273). Of the IFX cohort, 69 patients (74.2%) were biological-naïve at the start of the treatment. A total of 89 patients (95.7%) achieved induction, and 72 patients (77.4%) achieved the 1-year mark. A total of 165 patients were treated with ADA with a median of 1051 (48–4458) days on the medication. Of all patients who started ADA, 133 patients (80.6%) were not previously exposed to a biological treatment. A total of 165 (100%) patients achieved induction, and 133 patients (80.1%) achieved the 1-year mark.

A total of 116 patients were treated with VDZ with a median of 717 (0–2204) days on the medication. A total of 67 patients (58%) were biological-naïve at the start of treatment; 115 patients (99.1%) achieved induction; 82 patients (70.7%) achieved the 1-year mark.

**Figure 1:**
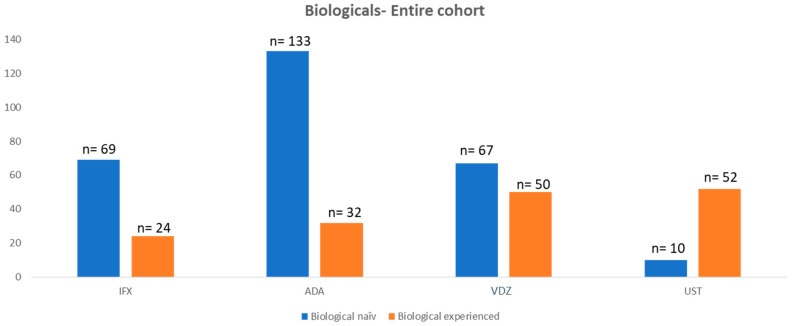
Number of patients with biological treatment of the entire cohort.

Lastly, 62 patients were treated with UST with a median duration of 660 days (50–1399). A total of 60 patients (98.4%) achieved induction. Interestingly, only nine patients (14%) were not previously exposed to another biological agent prior to starting UST. A total of 44 patients (71%) achieved the 1-year mark (Table 2 and Figure 1)

When treated as a first-line biologic, UST (median 787 days) and VDZ (median 756.5 days) have the shortest treatment duration.

We examined the data to determine which first-line therapy was continued the longest before a switch in medication occurred due to loss of response or adverse effects. The TNF-α blockers showed the highest therapeutic resistance in this regard (ADA vs. VDZ *p* < 0.001, IFX vs. VDZ *p* < 0.001, Kruskal–Wallis test pairwise comparisons).

**Figure 2:**
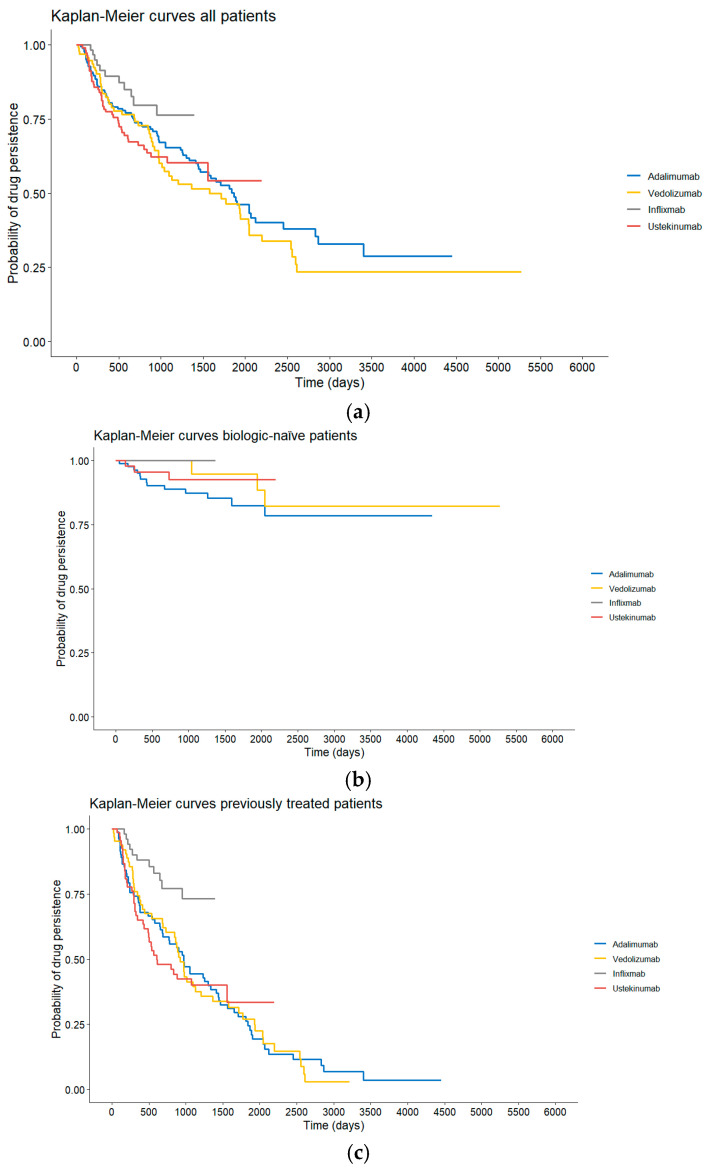
(**a**) Drug persistence of the different treatment groups of the entire cohort. (**b**) Drug persistence of the different treatment groups of biologic-naïve patients. (**c**) Drug persistence of the different treatment groups of biologic-experienced patients.

Cox regression analysis of the time until the change in medication (indifferent whether the drug was given as a first drug or not) included data of patients who received at least one of the four drugs. No evidence against the assumption of proportional hazards was found (*p* = 0.063). The analysis revealed significant differences between UST and VDZ (*p* = 0.011, HR = 0.41, 95% CI: (0.21, 0.81)) and IFX and VDZ (*p* = 0.004, HR = 0.49, 95% CI: (0.30, 0.80)) indicating a significantly higher persistence for VDZ compared to UST and IFX when accounting for confounders. Kaplan–Meier curves for all patients and stratified by biologic-naïve status are presented in Figure 2.

Sensitivity analysis using imputed data of the patients who received at least one of the four drugs (280 patients, 436 observations) confirmed the significant effects of treatment (UST vs. VDZ and IFX vs. VDZ) with comparable HRs and 95% confidence intervals (UST vs. VDZ:HR = 0.41, 95% CI: (0.22, 0.76) and IFX vs. VDZ:HR = 0.53, 95% CI: (0.34, 0.83)). The sensitivity analysis revealed a further significant difference between ADA and VDZ (*p* = 0.014, HR = 0.58, 95% CI: (0.36, 0.89)). This hazard ratio is comparable to the one from the complete-case analysis (HR = 0.64, 95% CI: (0.38, 1.06)) that included 366 observations from 252 patients with complete data for the confounders.

### 3.3. Steroids at Baseline per Treatment and Disease Entity

The need for steroids at baseline was evaluated for all treatment groups. Although we observed no statistically significant differences between the treatment groups, the need for steroids was slightly higher in patients diagnosed with UC (Table 3).

### 3.4. Fecal Calprotectin as a Parameter for Disease Activity

We assessed fCal for the entire cohort as well as for the respective disease entities. FCal values were obtained at baseline, at 6 months, and after 1 year. Due to the retrospective nature of this study, results were not available for all patients. We observed a significant reduction in fCal between baseline and the 6-month as well as the 1-year time-point for the entire cohort as well as for the respective disease entity subgroups. At baseline, fCal values were increased above 250 µg/g (defined as the discriminator between high/low disease activity) throughout all treatment groups. After 6 months, fCal values were below 250 µg/g except for patients with CD in the IFX group (Table 4). After 1 year, values were below 250 µg/g in all groups.

The mixed-effects linear regression showed a significant decrease of 2% in average per month of the fCal values (*p* < 0.001). FCal values of patients with UC were significantly lower (by estimated 7%) compared to those with CD (*p* = 0.044). The effect of treatment was not significant. The complete case model included 459 observations from 190 patients (conditional R^2^ = 0.432, marginal R^2^ = 0.135).

The significant decrease in fCal with time and the significant lower values for patients with UC were confirmed in the sensitivity analysis. Furthermore, the directions of the associations between fCal and treatment were confirmed and also proved to be significant in the sensitivity analysis which included 1183 observations from 280 patients. Since the size of the imputed dataset for this analysis was 2.5 times larger than the complete-case dataset, this is not surprising. Nevertheless, the result should be interpreted with care.

When compared with the drug-persistence rates, the response rates were lower for IFX, ADA, and UST. Interestingly, for VDZ, the response rates were higher than the drug-persistence rates (83% vs. 71%). An explanation might be that patients in the VDZ group in general had lower fCal values at all time-points.

## 4. Discussion

Drug persistence is becoming an increasingly important factor in the management of IBD treatment. It is a parameter that is easy to assess and provides indirect insight into treatment efficacy. Moreover, patients often inquire about treatment persistence rates, particularly when they have already undergone multiple therapeutic regimens. Despite its significance, data on this topic remain limited, and further critical examination is needed to understand the factors influencing persistence and its implications for long-term disease management.

**In our study**, we found that the majority of patients continued on the same medication after one year. Additionally, there was a reduction in fCal levels—a marker of inflammatory activity—over the course of the year, and corticosteroid use could be decreased. While these findings are promising, they warrant further exploration, particularly in terms of how these outcomes relate to treatment satisfaction, patient adherence, and real-world disease management.

In this cohort, treatment persistence differed notably among the evaluated biologic therapies. When used as first-line agents, UST and VDZ showed the shortest median treatment durations, suggesting earlier discontinuation compared with TNF-α inhibitors. Consistently, first-line TNF-α blockers demonstrated the longest continuation times before treatment failure or adverse events prompted a switch, which was supported by significantly longer persistence compared with VDZ in pairwise analyses. This may be explained by the fact that in our clinic we perform regular drug monitoring and ensure that patients maintain adequate medication trough levels.

However, when adjusting for confounders in the Cox regression analysis, VDZ showed significantly higher overall persistence than UST and IFX, indicating that its treatment duration advantage emerges when factors beyond simple chronological drug order are taken into account. The proportional hazards assumption was met, strengthening the validity of the model. Sensitivity analyses using imputed data confirmed these associations, further identifying a significant difference between ADA and VDZ that was not statistically significant in the complete-case dataset but showed a comparable effect size.

Taken together, these findings highlight the complexity of evaluating biologic durability in real-world practice. While TNF-α inhibitors appear most persistent when used as initial therapy, VDZ demonstrates superior persistence once patient characteristics and treatment histories are incorporated. This underscores the importance of adjusting for confounding variables when comparing biologic performance.

Several studies have investigated drug persistence in both treatment-naïve and experienced patients. The VARSITY trial, for example, compared VDZ versus ADA for patients with moderate-to-severe UC and reported VDZ to be superior to ADA with respect to endoscopic healing and clinical remission but not steroid-free clinical remission [22]. However, this trial’s patient population is not fully representative of those in everyday clinical settings, as most routine patients would not meet the eligibility criteria for a randomized clinical trial [23]. This discrepancy highlights the need for real-world data to draw more realistic conclusions about drug persistence and its implications.

The VEDO IBD trial, which investigated treatment-naïve patients with UC, found that VDZ resulted in higher remission rates than anti-TNF agents after two years of treatment [24]. In a second trial, patients with CD were analyzed. The results of this prospective, 2-year, real-world evidence study suggest that the choice of VDZ led to higher remission rates after 2 years compared with anti-TNF. This could support the role of VDZ as a first-line biologic therapy in CD [25].

A retrospective study by Moens et al. [26] aimed to translate findings from the VARSITY trial into a real-world setting by investigating biologic-naïve IBD patients starting either VDZ or ADA. They found that VDZ was superior in terms of treatment persistence and endoscopic remission for patients with UC at week 52, but not for patients with CD. In contrast, our study found that anti-TNF agents (IFX and ADA) were superior to VDZ in terms of treatment persistence (*p* < 0.001). This discrepancy suggests that factors such as disease severity, patient population, and baseline treatment history may significantly influence the outcomes, and more targeted research is required to understand these dynamics.

Chen et al. [27] provided further real-world data on treatment persistence in over 9000 newly diagnosed IBD patients in the United States (US). Their study reported that less than 50% of patients continued their initial biological treatment after one year. These rates are substantially lower than those observed in our cohort, which might be attributed to the inclusion of both treatment-naïve and treatment-experienced patients. In addition, our cohort had a longer median disease duration compared to the US cohort, suggesting that longer disease duration may correlate with higher persistence due to more established treatment plans or greater disease control over time.

A Swedish registry study, which evaluated drug persistence for anti-TNF agents and VDZ as second-line biological treatments in IBD patients, found similar effectiveness and safety profiles for both medications at 12 months [28]. Drug persistence for anti-TNF after one year was reported to be 74% for patients with CD and 62% for patients with UC. In our cohort, drug persistence was also above 70% for both anti-TNF agents and for both disease entities at this time-point. Specifically, more patients with UC remained on IFX after one year (81%) compared to patients with CD (76%), while the opposite trend was observed for ADA (CD 84% vs. UC 71%). Although the differences were not statistically significant, the observed trends suggest that disease type and biologic choice may influence persistence rates. As we included both treatment-naïve and treatment-exposed patients, therefore our results are not entirely comparable.

This difference could reflect inherent disease characteristics or the impact of including both treatment-naïve and treatment-experienced patients in our study, making direct comparisons with other cohorts challenging.

An observational retrospective study by Lukin et al. [29] included both treatment-naïve and -experienced UC patients treated with either VDZ or TNF-antagonists. Their propensity score-matched cohort found that patients treated with VDZ were more likely to achieve remission than those on TNF-antagonists. However, comparing this result with ours is difficult because our study did not use propensity score matching and did not assess remission through clinical scores or endoscopic reports, leaving some of the nuanced benefits of treatment unclear.

Additionally, we included data on treatment persistence for UST in patients with CD. A nationwide real-world study from Finland investigating UST persistence in 155 patients with CD reported that 83% of patients remained on UST after more than one year [30]. Another long-term study from Spain, which included 463 patients with CD, showed that 77% continued treatment after 15 months. Our study reported a similar persistence rate of 71%, although slightly lower. This difference could reflect variations in patient populations, disease severity, and treatment protocols across studies.

Our study has several limitations. Foremost is that this is a retrospective observational study conducted at a tertiary center and may therefore not entirely reflect the routine practice. 

This selection bias could affect the generalizability of our results, and caution should be exercised when applying these findings to broader IBD populations. Furthermore, the retrospective nature of the study limits our ability to control for all potential confounding factors. While we attempted to account for known variables, unmeasured factors (e.g., patient adherence, specific disease features, or comorbidities) may still introduce bias into our analysis. Future prospective, multi-center studies are needed to validate our findings in more diverse and representative cohorts.

Still, our observations appear to be similar with the results from the VARSITY Trial as well as from some of the real-world studies on drug persistence [22,28].

A further limitation is that, due to the data collection period, we did not analyze any data on the treatment with UST in UC, as the drug has only been approved in Europe since 2019 [13]. However, a Swedish study also found a similar persistence rate in UC as in CD, but with an observation period of only 16 weeks [31]. Another real-world study compared UST with ADA in UC and showed patients receiving UST had higher persistence after 12 months of treatment (83.8% therapy-naïve patients and 78.1% therapy-experienced patients) [32].

Another limitation is the incomplete data on fCal values and concomitant steroid treatment. Due to the retrospective nature of our study, data on endoscopic and histological reports, disease activity scores, or reasons for treatment discontinuation were not available for analysis. Therefore, we cannot provide reliable data on reasons for treatment discontinuation or remission. Nonetheless, the rate of patients that continued treatment after the respective induction period was above 90% in all treatment groups in our cohort. The rate was equally high for the three-month mark, indirectly indicating a low rate of non-responders throughout the biological groups. Lastly, drug persistence does serve as an indirect parameter of treatment efficacy, assuming patients will not continue a medication if sufficient disease control is not achieved. Further studies with larger sample sizes are needed to confirm these observations.

Future persistence analyses should incorporate inflammatory-biomarker-guided approaches, as proposed in the Treat-SMART study (ECCO 2025), to align treatment duration with objective disease activity markers and enhance personalized therapeutic strategies [33].

## 5. Conclusions

Our study provides data on drug persistence for the most commonly used biological treatment regimens in both treatment-naïve and treatment-experienced patients with IBD. Although some statistically significant differences emerged in subgroup and adjusted analyses, overall persistence rates across the four biologic agents were broadly comparable in the real-world setting. This study contributes to the growing body of evidence on the long-term use of biologics in the IBD population.

Importantly, our results demonstrate that existing therapies continue to offer effective and cost-efficient benefits and remain highly relevant, even with the emergence of IL-23 inhibitors. 

Emerging delivery strategies and biosimilar formulations have reshaped biologic therapy, emphasizing the need to interpret persistence data in light of evolving targeting mechanisms and patient-centric approaches [34]. 

This information may support clinicians in guiding patients regarding the optimal positioning of specific therapies within the broader IBD treatment landscape.

## Figures and Tables

**Table 1:** Patient characteristics. (**a**) Vienna Classification. (**b**) Montreal Classification.

	Entire Cohort, n = 587	Crohn’s Disease, n = 312 (53%)	Ulcerative Colitis, n = 275 (47%)
Age years, median (range)	44 (20–87)	42 (20–87)	46 (20–87)
Female gender, n (%)	311 (53)	185 (59.2)	126 (45.8)
Disease duration years, n = 576 median (range)	14 (1–52)	n= 304, 15 (1–52)	n = 272, 11.5 (1–50)
(**a**)			
		**Vienna Classification**	
A n		294/312	
A1 n (%)		247 (84)	
A2 n (%)		45 (15.3)	
A3 n (%)		2 (0.7)	
L n		289/312	
L1 n (%)		89 (30.8)	
L2 n (%)		56 (19.3)	
L3 n (%)		111 (38.4)	
L4 n (%)		33 (11.4)	
B n		277/312	
B1 n (%)		122 (44)	
B2 n (%)		59 (21.3)	
B3 n (%)		96 (34.6)	
(**b**)			
		**Montreal Classification 241/275**	
**1** n (%)		36 (14.9)	
**2** n (%)		77 (32)	
**3** n (%)		128 (53.1)	

**Table 2:** Drug persistence of the different biological treatments.

	Entire Subcohort, n = 280	Crohn’s Disease, n = 184, (66%)	Ulcerative Colitis, n = 96, (34%)
IFX, n (%)	93 (33.21)	67 (36.41)	26 (27.08)
Induction, n (%)	89 (95.7)	64 (95.5)	25 (96.2)
3 months, n (%)	89 (95.7)	65 (97)	25 (96.2)
6 months, n (%)	86 (92.5)	63 (94)	23 (88.5)
1 year, n (%)	72 (77.4)	51 (76.1)	21 (80.8)
Time on med, days median (range)	912 (20–5273)	924 (20–4796)	865 (35–5273)
ADA, n (%)	165 (58.93)	122 (66.30)	44 (45.83)
Induction, n (%)	165 (100)	122 (100)	44 (100)
3 months, n (%)	162 (98.2)	122 (100)	40 (90.9)
6 months, n (%)	149 (90.3)	115 (94.3)	34 (77.3)
1 year, n (%)	133 (80.6)	102 (83.6)	31 (70.5)
Time on med, days median (range)	1051 (48–4458)	1219 (90–4458)	909 (48–3088)
VDZ, n (%)	116 (41.43)	67 (36.41)	49 (52.13)
Induction, n (%)	115 (99.1)	66 (98.5)	49 (100)
3 months, n (%)	112 (96.6)	65 (97)	47 (96)
6 months, n (%)	100 (86.2)	54 (80.6)	46 (93.8)
1 year, n (%)	82 (70.7)	43 (64.2)	39 (79.6)
Time on med, days median (range)	717 (0–220)	554 (0–2194)	860 (48–2200)
UST, n (%)	62 (22.14)	59 (30.07)	/
Induction, n (%)	60 (96.8)	58 (98.3)	/
3 months, n (%)	60 (96.8)	58 (98.3)	/
6 months, n (%)	57 (91.9)	55 (93.2)	/
1 year, n (%)	44 (71)	44 (74.6)	/
Time on med, days median (range)	660 (50–1399)	676 (50–1399)	/

Infliximab (IFX), Adalimumab (ADA), Vedolizumab (VDZ), Ustekinumab (UST).

**Table 3:** Steroids at baseline per treatment and disease entity.

	Entire Subcohort, n = 280	Crohn’s Disease, n = 184, (66%)	Ulcerative Colitis, n = 96, (34%)	*p*-Value (CHI Quadrat Test)
IFX	93	67	26	
Steroids at baseline, n (%)	34 (36.6)	25 (37.3)	9 (34.6)	0.63
ADA	165	122	44	
Steroids at baseline, n (%)	56 (33.9)	35 (28.7)	21 (47.7)	0.11
VDZ	116	67	49	
Steroids at baseline, n (%)	50 (43.1)	27 (40.3)	23 (46.9)	0.95
UST	62	59	/	
Steroids at baseline, n (%)	25 (40.3)	23 (39)	/	

Infliximab (IFX), Adalimumab (ADA), Vedolizumab (VDZ), Ustekinumab (UST).

**Table 4:** Fecal Calprotectin over time and by disease entity.

	Entire Subcohort, n = 280	Crohn’s Disease, n = 184, (66%)	Ulcerative Colitis, n = 96, (34%)
IFX			
fCal at baseline, n median (Range)	n= 37, 473 (51–1935)	n = 24, 455 (68–1778)	n = 13, 473 (51–1935)
fCal at 6 months, n median (Range)	n = 28, 242 (12–1544)	n = 18, 280 (35–1544)	n = 10, 97 (12–1073)
fCal at 1 year, n median (Range)	n = 26, 180 (17–697)	n = 16, 205 (15–697)	n = 10, 145 (18–304)
ADA			
fCal at baseline, n median (Range)	n = 72, 424 (23–1905)	n = 54, 406.5 (23–1905)	n = 18, 457 (55–1360)
fCal at 6 months, n median (Range	n = 50, 118 (11–1328)	n = 36, 118.5 (12–1328)	n = 14, 167.5 (11–1283)
fCal at 1 year, n median (Range	n = 41, 128 (15–1840)	n = 28, 188 (22–1840)	n = 13, 92 (15–615)
VDZ			
fCal at baseline, n median (Range	n = 55, 275 (3–1973)	n = 28, 281 (47–1430)	n = 27, 231 (3–1973)
fCal at 6 months, n median (Range	n = 55, 118 (12–1923)	n = 24, 175 (12–1614)	n = 31, 105 (13–1923)
fCal at 1 year, n median (Range)	n = 42, 73 (15–1222)	n = 25, 73 (19–1222)	n = 17, 84 (15–468)
UST			
fCal at baseline, n median (Range)	n = 26, 302 (20–2078)	n = 24, 302.5 (20–2078)	
fCal at 6 months, n median (Range)	n = 19, 137 (8–1476)	n = 17, 137 (8–1476)	
fCal at 1 year, n median (Range)	n = 19, 117 (8–1248)	n = 19, 117 (8–1248)	

Infliximab (IFX), Adalimumab (ADA), Vedolizumab (VDZ), Ustekinumab (UST), fecal Calprotectin (fCal).

## Data Availability

The original contributions presented in this study are included in the article. Further inquiries can be directed to the corresponding author.

## References

[1] Hanauer S.B., Feagan B.G., Lichtenstein G.R., Mayer L.F., Schreiber S., Colombel J.F., Rachmilewitz D., Wolf D.C., Olson A., Bao W. (2002). Maintenance infliximab for Crohn’s disease: The ACCENT I randomised trial. Lancet.

[2] Targan S.R., Hanauer S.B., van Deventer S.J., Mayer L., Present D.H., Braakman T., DeWoody K.L., Schaible T.F., Rutgeerts P.J. (1997). A short-term study of chimeric monoclonal antibody cA2 to tumor necrosis factor alpha for Crohn’s disease. Crohn’s Disease cA2 Study Group. N. Engl. J. Med..

[3] Rutgeerts P., Sandborn W.J., Feagan B.G., Reinisch W., Olson A., Johanns J., Travers S., Rachmilewitz D., Hanauer S.B., Lichtenstein G.R. (2005). Infliximab for induction and maintenance therapy for ulcerative colitis. N. Engl. J. Med..

[4] Hanauer S.B., Sandborn W.J., Rutgeerts P., Fedorak R.N., Lukas M., MacIntosh D., Panaccione R., Wolf D., Pollack P. (2006). Human anti-tumor necrosis factor monoclonal antibody (adalimumab) in Crohn’s disease: The CLASSIC-I trial. Gastroenterology.

[5] Sandborn W.J., Hanauer S.B., Rutgeerts P., Fedorak R.N., Lukas M., MacIntosh D.G., Panaccione R., Wolf D., Kent J.D., Bittle B. (2007). Adalimumab for maintenance treatment of Crohn’s disease: Results of the CLASSIC II trial. Gut.

[6] Reinisch W., Sandborn W.J., Hommes D.W., D’Haens G., Hanauer S., Schreiber S., Panaccione R., Fedorak R.N., Tighe M.B., Huang B. (2011). Adalimumab for induction of clinical remission in moderately to severely active ulcerative colitis: Results of a randomised controlled trial. Gut.

[7] Sandborn W.J., van Assche G., Reinisch W., Colombel J.F., D’Haens G., Wolf D.C., Kron M., Tighe M.B., Lazar A., Thakkar R.B. (2012). Adalimumab induces and maintains clinical remission in patients with moderate-to-severe ulcerative colitis. Gastroenterology.

[8] Colombel J.F., Sandborn W.J., Rutgeerts P., Enns R., Hanauer S.B., Panaccione R., Schreiber S., Byczkowski D., Li J., Kent J.D. (2007). Adalimumab for maintenance of clinical response and remission in patients with Crohn’s disease: The CHARM trial. Gastroenterology.

[9] Rutgeerts P., Feagan B.G., Marano C.W., Padgett L., Strauss R., Johanns J., Adedokun O.J., Guzzo C., Zhang H., Colombel J.F. (2015). Randomised clinical trial: A placebo-controlled study of intravenous golimumab induction therapy for ulcerative colitis. Aliment. Pharmacol. Ther..

[10] Eriksson C., Marsal J., Bergemalm D., Vigren L., Bjork J., Eberhardson M., Karling P., Soderman C., Group S.V.S., Myrelid P. (2017). Long-term effectiveness of vedolizumab in inflammatory bowel disease: A national study based on the Swedish National Quality Registry for Inflammatory Bowel Disease (SWIBREG). Scand. J. Gastroenterol..

[11] Shelton E., Allegretti J.R., Stevens B., Lucci M., Khalili H., Nguyen D.D., Sauk J., Giallourakis C., Garber J., Hamilton M.J. (2015). Efficacy of Vedolizumab as Induction Therapy in Refractory IBD Patients: A Multicenter Cohort. Inflamm. Bowel Dis..

[12] Vivio E.E., Kanuri N., Gilbertsen J.J., Monroe K., Dey N., Chen C.H., Gutierrez A.M., Ciorba M.A. (2016). Vedolizumab Effectiveness and Safety Over the First Year of Use in an IBD Clinical Practice. J. Crohns Colitis.

[13] Sands B.E., Sandborn W.J., Panaccione R., O’Brien C.D., Zhang H., Johanns J., Adedokun O.J., Li K., Peyrin-Biroulet L., Van Assche G. (2019). Ustekinumab as Induction and Maintenance Therapy for Ulcerative Colitis. N. Engl. J. Med..

[14] Feagan B.G., Sandborn W.J., Gasink C., Jacobstein D., Lang Y., Friedman J.R., Blank M.A., Johanns J., Gao L.L., Miao Y. (2016). Ustekinumab as Induction and Maintenance Therapy for Crohn’s Disease. N. Engl. J. Med..

[15] Van den Berghe N., Verstockt B., Tops S., Ferrante M., Vermeire S., Gils A. (2019). Immunogenicity is not the driving force of treatment failure in vedolizumab-treated inflammatory bowel disease patients. J. Gastroenterol. Hepatol..

[16] Allez M., Karmiris K., Louis E., Van Assche G., Ben-Horin S., Klein A., Van der Woude J., Baert F., Eliakim R., Katsanos K. (2010). Report of the ECCO pathogenesis workshop on anti-TNF therapy failures in inflammatory bowel diseases: Definitions, frequency and pharmacological aspects. J. Crohns Colitis.

[17] Ben-Horin S., Kopylov U., Chowers Y. (2014). Optimizing anti-TNF treatments in inflammatory bowel disease. Autoimmun. Rev..

[18] Danese S., Fiorino G., Reinisch W. (2011). Review article: Causative factors and the clinical management of patients with Crohn’s disease who lose response to anti-TNF-α therapy. Aliment. Pharmacol. Ther..

[19] Gasche C., Scholmerich J., Brynskov J., D’Haens G., Hanauer S.B., Irvine E.J., Jewell D.P., Rachmilewitz D., Sachar D.B., Sandborn W.J. (2000). A simple classification of Crohn’s disease: Report of the Working Party for the World Congresses of Gastroenterology, Vienna 1998. Inflamm. Bowel Dis..

[20] Silverberg M.S., Satsangi J., Ahmad T., Arnott I.D., Bernstein C.N., Brant S.R., Caprilli R., Colombel J.F., Gasche C., Geboes K. (2005). Toward an integrated clinical, molecular and serological classification of inflammatory bowel disease: Report of a Working Party of the 2005 Montreal World Congress of Gastroenterology. Can. J. Gastroenterol..

[21] Mowat C., Cole A., Windsor A., Ahmad T., Arnott I., Driscoll R., Mitton S., Orchard T., Rutter M., Younge L. (2011). Guidelines for the management of inflammatory bowel disease in adults. Gut.

[22] Sands B.E., Peyrin-Biroulet L., Loftus E.V., Danese S., Colombel J.F., Toruner M., Jonaitis L., Abhyankar B., Chen J., Rogers R. (2019). Vedolizumab versus Adalimumab for Moderate-to-Severe Ulcerative Colitis. N. Engl. J. Med..

[23] Ha C., Ullman T.A., Siegel C.A., Kornbluth A. (2012). Patients enrolled in randomized controlled trials do not represent the inflammatory bowel disease patient population. Clin. Gastroenterol. Hepatol..

[24] Bokemeyer B., Plachta-Danielzik S., di Giuseppe R., Efken P., Mohl W., Krause T., Hoffstadt M., Ehehalt R., Trentmann L., Schweitzer A. (2023). Real-world effectiveness of vedolizumab compared to anti-TNF agents in biologic-naïve patients with ulcerative colitis: A two-year propensity-score-adjusted analysis from the prospective, observational VEDO_IBD_-study. Aliment. Pharmacol. Ther..

[25] Bokemeyer B., Plachta-Danielzik S., di Giuseppe R., Efken P., Mohl W., Hoffstadt M., Krause T., Schweitzer A., Schnoy E., Atreya R. (2024). Real-World Effectiveness of Vedolizumab vs Anti-TNF in Biologic-naive Crohn’s Disease Patients: A 2-year Propensity-score-adjusted Analysis from the VEDOIBD-Study. Inflamm. Bowel Dis..

[26] Moens A., Verstockt B., Alsoud D., Sabino J., Ferrante M., Vermeire S. (2022). Translating Results from VARSITY to Real World: Adalimumab vs Vedolizumab as First-line Biological in Moderate to Severe IBD. Inflamm. Bowel Dis..

[27] Chen C., Hartzema A.G., Xiao H., Wei Y.J., Chaudhry N., Ewelukwa O., Glover S.C., Zimmermann E.M. (2019). Real-world Pattern of Biologic Use in Patients With Inflammatory Bowel Disease: Treatment Persistence, Switching, and Importance of Concurrent Immunosuppressive Therapy. Inflamm. Bowel Dis..

[28] Rundquist S., Sachs M.C., Eriksson C., Olen O., Montgomery S., Halfvarson J., Group S.S. (2021). Drug survival of anti-TNF agents compared with vedolizumab as a second-line biological treatment in inflammatory bowel disease: Results from nationwide Swedish registers. Aliment. Pharmacol. Ther..

[29] Lukin D., Faleck D., Xu R., Zhang Y., Weiss A., Aniwan S., Kadire S., Tran G., Rahal M., Winters A. (2022). Comparative Safety and Effectiveness of Vedolizumab to Tumor Necrosis Factor Antagonist Therapy for Ulcerative Colitis. Clin. Gastroenterol. Hepatol..

[30] Af Bjorkesten C.G., Ilus T., Hallinen T., Soini E., Eberl A., Hakala K., Heikura M., Jussila A., Koskela R., Koskinen I. (2020). Objectively assessed disease activity and drug persistence during ustekinumab treatment in a nationwide real-world Crohn’s disease cohort. Eur. J. Gastroenterol. Hepatol..

[31] Thunberg J., Bjorkqvist O., Hedin C.R.H., Forss A., Soderman C., Bergemalm D., Group S.S., Olen O., Hjortswang H., Strid H. (2022). Ustekinumab treatment in ulcerative colitis: Real-world data from the Swedish inflammatory bowel disease quality register. United Eur. Gastroenterol. J..

[32] Zhdanava M., Kachroo S., Boonmak P., Burbage S., Shah A., Korsiak J., Lefebvre P., Kerner C., Pilon D. (2024). Real-World Treatment Persistence Among Advanced Therapy-Naive or -Experienced Patients with Ulcerative Colitis Initiated on Ustekinumab or Adalimumab. Adv. Ther..

[33] Medhat M., Ramadan Y.N., Hashem M., Doaa A., Nariman Z., Hetta H.F., Group” A.T.I.S. (2025). P1045 Treat-smart study: Leveraging individual inflammatory markers for personalized therapy in patients with IBD. J. Crohn’s Colitis.

[34] Aljabri A., Soliman G.M., Ramadan Y.N., Medhat M.A., Hetta H.F. (2025). Biosimilars versus biological therapy in inflammatory bowel disease: Challenges and targeting strategies using drug delivery systems. Clin. Exp. Med..

